# Promoting engagement with quality communication in social media

**DOI:** 10.1371/journal.pone.0275534

**Published:** 2022-10-13

**Authors:** Matteo Cinelli, Antonio Peruzzi, Ana Lucía Schmidt, Roberta Villa, Enrico Costa, Walter Quattrociocchi, Fabiana Zollo

**Affiliations:** 1 Ca’ Foscari University of Venice, Venice, Italy; 2 Sapienza University of Rome, Rome, Italy; University of Pisa, ITALY

## Abstract

The COVID-19 pandemic made explicit the issues of communicating science in an information ecosystem dominated by social media platforms. One of the fundamental communication challenges of our time is to provide the public with reliable content and contrast misinformation. This paper investigates how social media can become an effective channel to promote engagement and (re)build trust. To measure the social response to quality communication, we conducted an experimental study to test a set of science communication recommendations on Facebook and Twitter. The experiment involved communication practitioners and social media managers from select countries in Europe, applying and testing such recommendations for five months. Here we analyse their feedback in terms of adoption and show that some differences emerge across platforms, topics, and recommendation categories. To evaluate these recommendations’ effect on users, we measure their response to quality content, finding that the median engagement is generally higher, especially on Twitter. The results indicate that quality communication strategies may elicit positive feedback on social media. A data-driven and co-designed approach in developing counter-strategies is thus promising in tackling misinformation.

## Introduction

The COVID-19 pandemic has highlighted the challenge of conveying and communicating complexity and uncertainty [1]. This is especially true when considering the increasingly central role of the Internet and social media, that have deeply transformed the information landscape, leading to a shift away from a traditional content production paradigm. Designed to maximise users’ presence on the platform and to deliver targeted advertising, social media have rapidly become the main information sources for many of their users. Science communication has not been exempted from the changes introduced by this paradigm shift, with scientists and science institutions embracing public communication in the online environment [2]. Information spreads faster and farther online, in a flow-through system where users have immediate access to unlimited content. This may facilitate the proliferation of misinformation, generating chaos and limiting access to correct information.

Furthermore, individuals tend to process information, including scientific evidence, coherently with their system of beliefs [3, 4]. Online users have been shown to tend to aggregate in echo chambers, i.e., polarized groups of like-minded people [5, 6]. Immersed in these communities, users tend to ignore information dissenting from their worldview, even when evidence-based [7, 8]. This might make correction attempts ineffective, and in certain cases induce a backfire effect [9–11]. Additionally, when polarization is strong, misinformation might easily proliferate [12, 13]. These dynamics may take on great importance in the construction of social perceptions, as well as in the evolution of public debate on scientific issues, especially when disputed or controversial [14].

A crucial challenge is to improve the effectiveness and outreach of science communication [15, 16], especially in tackling the spread of misinformation [17, 18], science denialism [19, 20] and uncertainty of scientific advances [21, 22]. For this reason, important actors such as the National Academy of Sciences and the WHO have launched a series of actions aimed at empowering communication skills and strategies of science advocates [18, 23]. In addition, given the primary role of social media and online platforms in reaching and engaging with the public, communication has to be designed for online realms and corroborated by ad-hoc policies. Due to a combination of factors including information overload, users’ limited attention and feed algorithms, social media pose new challenges for tailoring effective communication strategies [24, 25].

Trying to understand how the digital age has changed science communication, many research works investigated the implications of online communication for science. In 2013 Brossard provided an overview of the new media landscape, focusing in particular on the bias introduced by search engines, as well as the characteristics of the scientific content available online and its potential impact on the users [2]. As pointed out by Davies and Hara [26], the analysis of available data reveals that the production and consumption of online content is complex, and requires further conceptualization. However, despite the wide availability of social media data, (quantitative) research into science communication on social media platforms is still scant [27, 28]. Moreover, little is known on what makes science communication on social media engaging, and communication practices are often “based on intuition and experiential rather than empirical evidence” [28]. To properly communicate complex (and controversial) science, the outcomes of online interactions with such content need to be studied in depth, and “this work has to be based on rigorous empirical social science rather than guesswork and anecdotal evidence” [29]. In this paper, we aim at offering a first, quantitative contribution in this direction, by addressing the following questions: RQ1) Can social media become an effective channel to promote engagement in science? RQ2) Can quality content positively engage users on social media?

To address these questions, we set up an innovative experiment through a co-design approach which involved science communication practitioners in select European countries. Our results show a positive increase in public engagement with science content. Overall, our work highlights that quality communication elicits a positive response in social media and highlights the benefits of a data-driven, co-designed approach in developing tailored counter-strategies, making a further step towards communications able to (re)build trust promote engagement, and contrast misinformation.

## Materials and methods

First, we designed a set of good practices (recommendations) for communicating science on social media, taking into account various aspects, from trustworthiness and scientific rigour, to presentation skills and societal impact, up to data-driven insights to develop tailored content. Then, we experimentally validated such recommendations with the direct involvement of science communication practitioners active on Facebook and Twitter. More specifically, a group of 22 science communication accounts and their social media managers received our set of recommendations and committed to test them in a period of five months.

### Recommendations for quality and effectiveness

To identify good practices for science communication, we started with a preliminary exploration of the landscape of science communication in social media in select European countries, as reported in [27]. To this aim, we collected and processed data from 498 Facebook pages and 661 Twitter accounts including a variety of sources –i.e., Festivals, Industries, Institutions, Magazines, Science Journalists, Experts, Scientists, and Universities. Our quantitative analysis of more than 2M tweets and posts across seven countries allowed to derive data-driven insights and identify patterns that appear to be associated with greater users’ engagement on social media. These results were enriched with opinions and feedback from diverse stakeholders (scholars, practitioners, journalists) on what quality in science communication means, and finally condensed into 12 quality indicators for science communication, with tailored suggestions for different social media platforms [30]. Starting from these indicators, we produced a set of recommendations to help communication practitioners such as science journalists, social media managers, universities, and organisations to improve both the quality of their content and its effectiveness, i.e., its impact in terms of engagement.

To extend the analysis to controversial issues, along with general recommendations, we also included more specific suggestions for three topics which are often linked to strong beliefs and emotions, i.e., vaccines [31–33], climate change [34–36], and artificial intelligence [37–40].

#### Quality

Quality criteria emerged by co-design activities (surveys, workshops, and interviews) organised with relevant stakeholders across select countries in Europe [30] and incorporate the perspective of both experts (journalists, communication practitioners, scientists) and the public on what constitutes quality in science communication. Three main conceptual areas were identified:

1. **Trustworthiness and scientific rigour**. This first area includes elements of quality connected to trust, which is fundamental in science communication [41]. Quality science communication does not only limit to provide reliable information, but also sheds light on the mechanisms behind knowledge creation and dissemination. In this direction, we identified the following quality criteria: 1) providing references to relevant scientific and/or official source(s); 2) fact-checking the content; 3) disclaiming one’s conflict of interest; and 4) promoting balanced communication, e.g., presenting comments from independent experts and key stakeholders, or reflect the diversity in the society, for example in terms of gender and other kinds of diversity.
2. **Presentation and style**. This second area includes a series of elements related to the quality of interaction with the public. These criteria focus on how scientific content is created and presented. The main challenge lies in being able to make science communication attractive without compromising its core values, i.e., trust, objectivity, transparency. In this direction, we provided the following recommendations: 1) using narrative and storytelling; 2) including *calls to actions*, e.g., asking questions, inviting to post or share content, organising flash mobs; 3) paying attention to the consistency and clarity of the message (e.g., between the text and the title); 4) ensuring that the length and complexity of sentences, the wording, and the assumptions are tailored for the target audience.
3. **Impact on society**. This third area focuses on the societal mission of science communication and how it can meet the audience’s needs and concerns. In this sense, the quality of communication depends on the capacity of acting as the main intermediary between science and society. We included the following recommendations: 1) addressing real life issues, e.g., by stressing the potential impact of scientific discoveries on daily life; 2) promoting positive changes in users’ behaviours (e.g., stop smoking, vaccinating, acting against climate change); 3) following ethical standards and considering social responsibility.

#### Effectiveness

As for effectiveness, preliminary quantitative results [27] revealed that specific content types may be associated with a higher engagement in terms of likes, comments and shares (Facebook), or of favourites and retweets (Twitter). The final set of recommendations can be summarised in what we have called “the 3Ts’ rule”, i.e., to always take into account 1) the Type of a tweet/post (post with only text, picture, video, link), 2) its Text (for example including hashtags or links), and 3) the Time of posting or tweeting during the day/week.

### Experimental setup

#### Recruitment of participants

To set up the experiment, we considered the list of social media accounts produced in in [27]. The list was created manually to represent a range of sources of science communication on social media across select countries in Europe as best as possible. Science communication is a fragmented and changing field, making it hard to obtain a representative sample. However, the list sought to include different categories of science communication sources (Science Festival, University, Industry/CEO, Science Journalist, Institution/Organization/Association, Magazine/Publication, Scientist/Expert) and to span across 7 countries (UK, Ireland, Italy, France, Germany, Estonia, Norway). We succeeded in involving 53 accounts listed in [27]. Unfortunately, the unexpected arrival of COVID-19 and its inevitable impact on work and personal lives made some of them withdraw their participation. Nonetheless, 27 social media accounts and their managers, whose contribution was voluntary and did not request any compensation for their activity, were actively involved in the experimental phase, with 22 of them constantly producing content following the recommendations.

By social media managers we mean the person (or group of people) who is in charge of the social media account. The term is not used here in reference to the job, indeed some of the accounts taking part in the experiment are managed by individuals (e.g., journalists, scientists) for which social media is not their primary working activity. In the experimental period, the social media manager(s) followed our suggestions when producing (some of the) content for their accounts, so that we could monitor users’ response to it. Furthermore, we organised online sessions during and after the experiment to discuss the clarity of our recommendations, as well as their validity and possible refinements. These discussions also proved to be fruitful to identify good practices on how to convey the complexity and uncertainty of science on social media platforms, especially during a historical event such as the COVID-19 pandemic. We integrated the participants’ feedback in the final version of our recommendations. Our interdisciplinary approach allowed us to co-design the recommendations with the help of those who will apply them in their daily activity on social media, thus maximising the likelihood of their future adoption among professionals. Participants were either active on both Twitter and Facebook (10) or on only one platform (9 on Twitter, 3 on Facebook). Each account is also associated with a topic label among General, Artificial Intelligence, Climate Change, and Vaccines according to its predominant subject. Table 1 provides a summary of the number of accounts involved in the experiment, as well as their main topic of discussion. By joining the experiment, the participants agreed to follow our recommendations when publishing science communication content in their Facebook and/or Twitter accounts in the period March-August 2020. Participants were free to select the content to publish and were expected to follow our recommendations for a total of 20 times (possibly) distributed along the whole five-month time period. Participants’ contribution to the experiment was voluntary and no compensation was provided for their activity.

**Table 1:** Summary of the number of accounts involved in the experimental phase on social media by topic.

Topic	Accounts
General	19
Climate Change	3
Artificial Intelligence	2
Vaccines	3

We assigned each account a code to track their social media activity and share with them a detailed document describing our recommendations. We also provided them with a checklist to facilitate their work and foster the adoption of our suggestions. Clearly, we did not expect that all items in the checklist were achieved simultaneously. Our advice was to follow the 3Ts rule, when possible, and to consider at least an element from the three aforementioned conceptual areas (trustworthiness and scientific rigour, presentation and style, impact on society). Participants were strongly encouraged to get in touch with the research team in case of any questions or doubts.

#### Data collection

1. **Self-reported data**. To monitor the effectiveness of the recommendations, we asked the participants to fill out a Google form to report on all the posts where they applied any of our recommendations. More specifically, the author of the post was asked to indicate the topic of the post (if any), if the content was fact checked or not, if gender balance was considered, and so on. The participants did not have any incentive to cheat, on the contrary they were actively involved in the experiment in a co-design activity. Therefore, it is reasonable to assume that the information reported in the form is trustworthy. The final dataset contains the following information: the code of the participant; the platform in which the post was produced (either Facebook or Twitter); the URL of the post/tweet; the topic of the post (which may differ from the topic label associated to the account); the recommendations followed when creating the post within the categories Trustworthiness and Scientific Rigour, Presentation and Style, and Impact and Style.
2. **Platform-specific API**. To compare the posts following the recommendations with another set of posts from the same participants, we downloaded the whole set of posts on the participants’ accounts during the period of observation that ranges from 27/02/2020 to 04/08/2020. The posts were downloaded using Twitter Standard APIs and the Facebook CrowdTangle service. In this case, we assume that the posts have the same topic label of the account that produced them. For example, an account labelled as Climate Change (thus posting mainly on the topic of climate change) could produce a post more focused on the use of artificial intelligence that will be however considered as a post about climate change. We downloaded 6,265 tweets from 19 Twitter accounts and 4,169 posts from 13 Facebook pages. The exact breakdown is provided in Tables 2 and 3, for Twitter and Facebook, respectively.

**Table 2:** Twitter. Count of posts, accounts and reactions to Twitter posts categorised by their compliance with the introduced recommendations.

		General	Artificial Intelligence	Climate Change	Vaccines	Total
Following Recomm.	Posts	130	14	62	34	240
	Accounts	14	2	1	2	19
	Favourites	2489	126	303	2476	5394
	Retweets	1073	52	139	619	1883
Not Following Recomm.	Posts	4765	47	875	578	6265
	Accounts	14	2	1	2	19
	Favourites	49120	138	1002	3494	53754
	Retweets	19767	66	538	1350	21721

**Table 3:** Facebook. Count of posts, accounts and reactions to Facebook posts categorised by their compliance with the introduced recommendations.

		General	Artificial Intelligence	Climate Change	Vaccines	Total
Following Recommendations	Posts	75	8	33	5	121
	Accounts	11	1	1	0	13
	Likes	6613	227	573	369	7782
	Comments	409	32	21	93	555
	Shares	2053	57	242	152	2504
Not Following Recommendations	Posts	4169	122	119	0	4410
	Accounts	11	1	1	0	13
	Likes	284430	1263	1146	0	286839
	Comments	40859	104	17	0	40980
	Shares	141254	152	354	0	141760

## Results

### Adoption rate

So far, we have described the principles employed for crafting the recommendations and recruiting the participants to the experiment. In this section, we analyse their adoption rate in the tweets and posts of active participants for the four selected categories, namely: General, Artificial Intelligence, Climate Change, and Vaccines. A breakdown of the recommendations is reported in Supplementary Information.

#### Twitter

**Fig 1:**
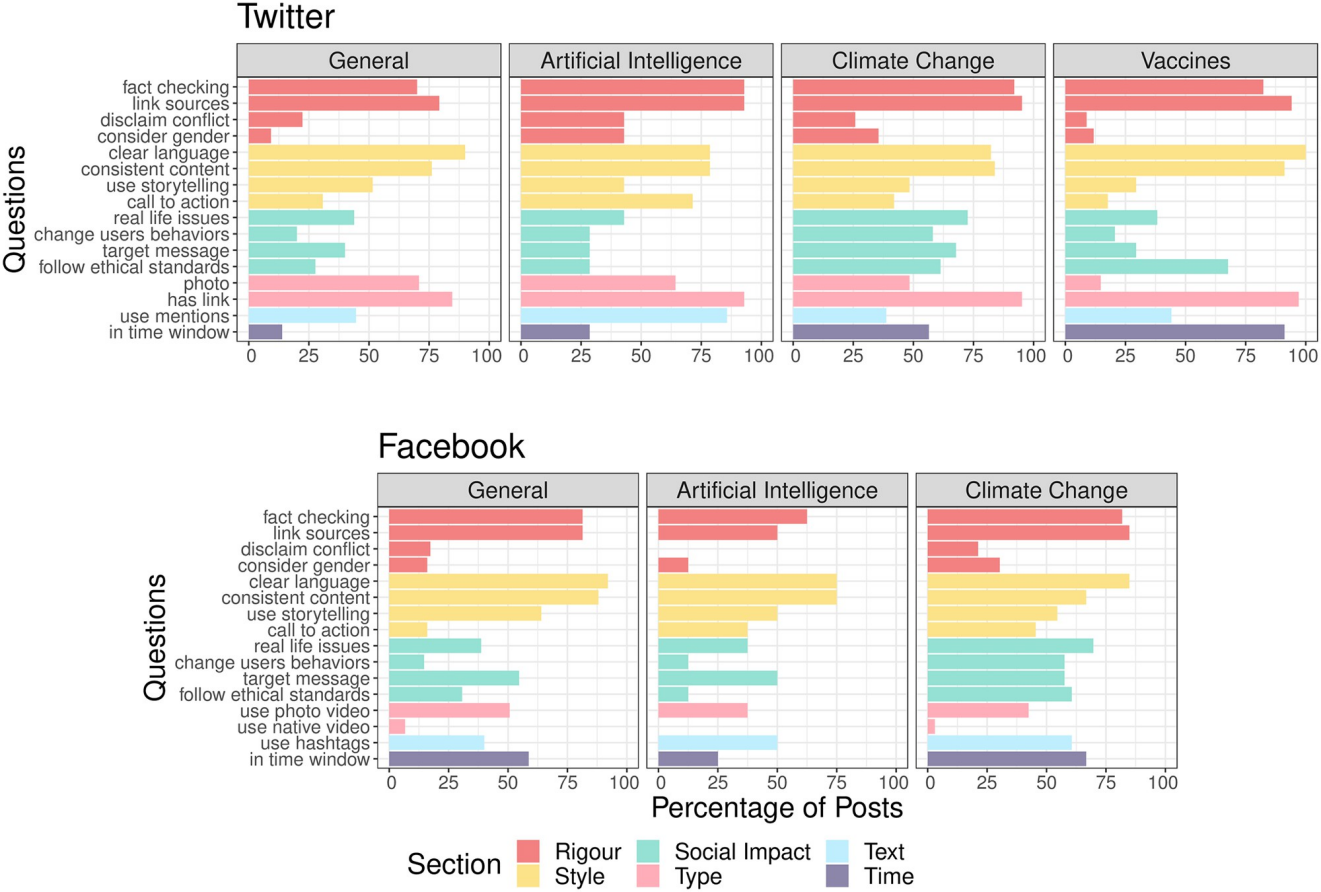
Adoption rates on social media platforms. Adoption rate of the recommendations for the considered topics, for Twitter (top) and Facebook (bottom). For Facebook, the topic Vaccines is missing due to the limited number of posts.

The number of tweets following the recommendations is 240, distributed across topics as follows: 130 posts on General, 14 on Artificial Intelligence, 62 on Climate Change, and 34 on Vaccines. Fig 1 shows that the Twitter accounts used all the recommendations. In particular, we observe that suggestions related to Rigour, Style and Type of posts are on average the most popular across topics. Other recommendations such as those related to Social Impact and to the time of posting had a more heterogeneous adoption rate. Especially the adoption of suggested posting times was strictly followed in the case of Vaccines, while this was not the case for General posts.

#### Facebook

A similar analysis was conducted for Facebook. In this case, the topic Vaccines is missing due to the limited number of posts (N = 5), which did not allow for a solid analysis. Fig 1 displays the adoption rate of the recommendations in Facebook posts of active participants for the three considered categories: Artificial Intelligence, Climate Change and General. The number of posts adopting the recommendations is 116, distributed across categories as follows: 8 posts on Artificial Intelligence, 33 posts on Climate Change, and 75 posts on General. As also observed in the case of Twitter, recommendations related to Rigour, Style and Type are on average the most popular across topics. Overall, the adoption rate of recommendations on Facebook is lower compared to Twitter, especially for Artificial Intelligence. Other recommendations such as those related to Social Impact and time of posting show instead a more heterogeneous adoption rate.

#### Joint adoption rate

In this section, we analyse the joint adoption rate of the recommendations, i.e., the co-occurrence of two recommendations, intended as the number of times two suggestions are jointly adopted in the same post. Investigating the set of co-occurrences of recommendations could be useful to unveil preferential mechanisms of joint adoption.

#### Twitter

**Fig 2:**
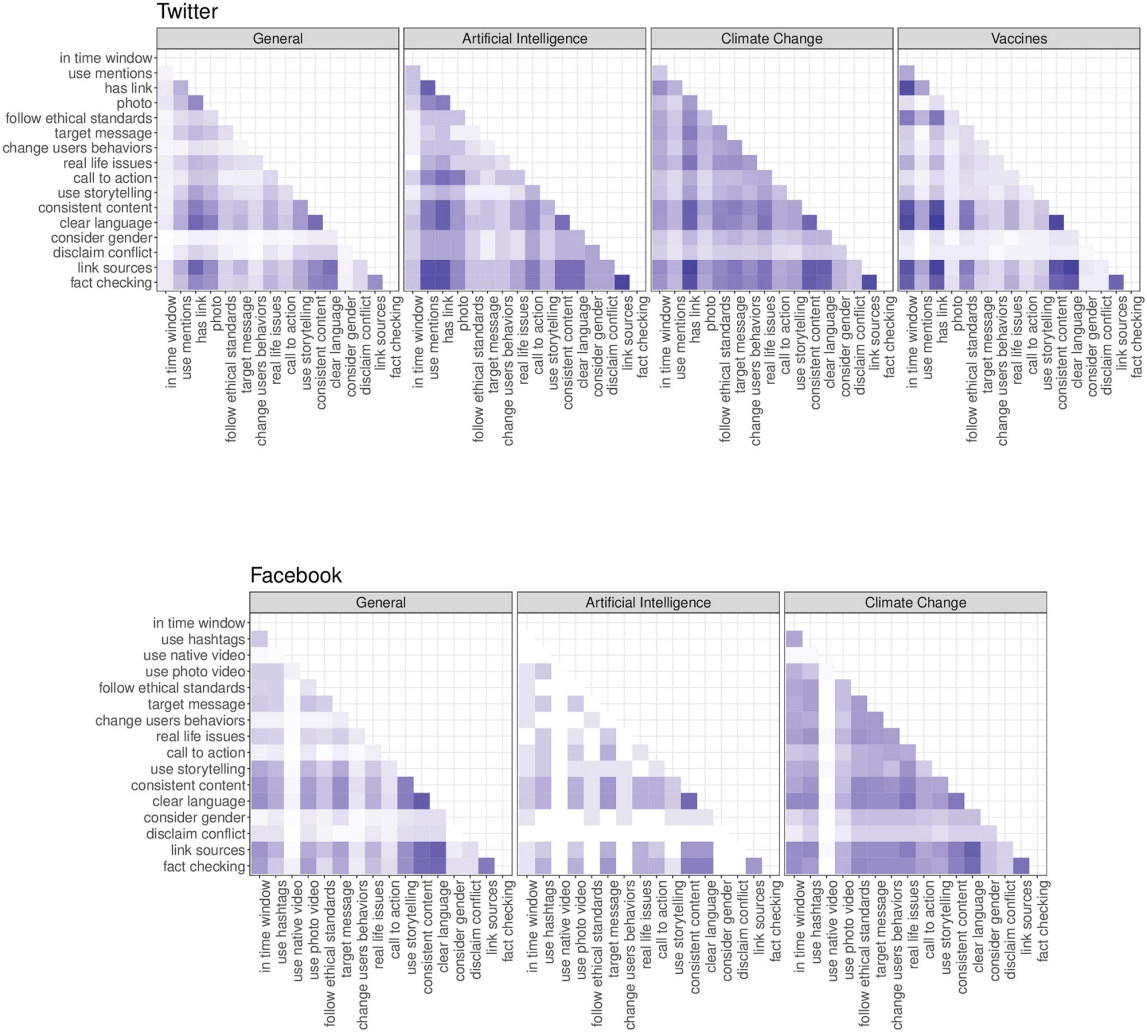
Co-occurrence of recommendation on social media platforms. Co-occurrence matrices displaying the proportion of recommendations applied together within the same posts for Twitter (top) and Facebook (bottom). Since the co-occurrence is a reciprocal quantity, the matrix is symmetric around the diagonal, thus only the upper triangular part is reported. As shown in the legend, darker colours indicate a higher co-occurrence of recommendations. In the case of Facebook, the topic Vaccines is missing due to the limited number of posts.

Fig 2 shows the results for Twitter. To obtain comparable proportions, the frequency of each co-occurrence is normalised by its maximum value, that is the number of posts per topic. Overall, we observe a relatively high proportion of co-occurrences (the average co-occurrence of two recommendations is 32.5% with a standard deviation of 22.6%) especially for Artificial Intelligence and Climate Change. Clearly, less employed recommendations are also less likely to co-occur in combination with others, e.g., in the case of “disclaim conflict” and “consider gender” in posts labelled as General and Vaccines.

#### Facebook

Fig 2 shows the co-occurrence of the recommendations for the three considered topics. The situation is different to what observed in Twitter, with a lower average co-occurrence of recommendations (the average co-occurrence is 24.3% with a standard deviation of 20.7%). Again, the results of co-occurrence of recommendations depend on the adoption rates displayed in Fig 1.

### Effectiveness

To evaluate the effectiveness of the proposed recommendations, we analyse the engagement of the posts that adopted them in comparison to the posts produced by the same accounts during the same time window, in which recommendations were not considered.

#### Twitter

**Fig 3:**
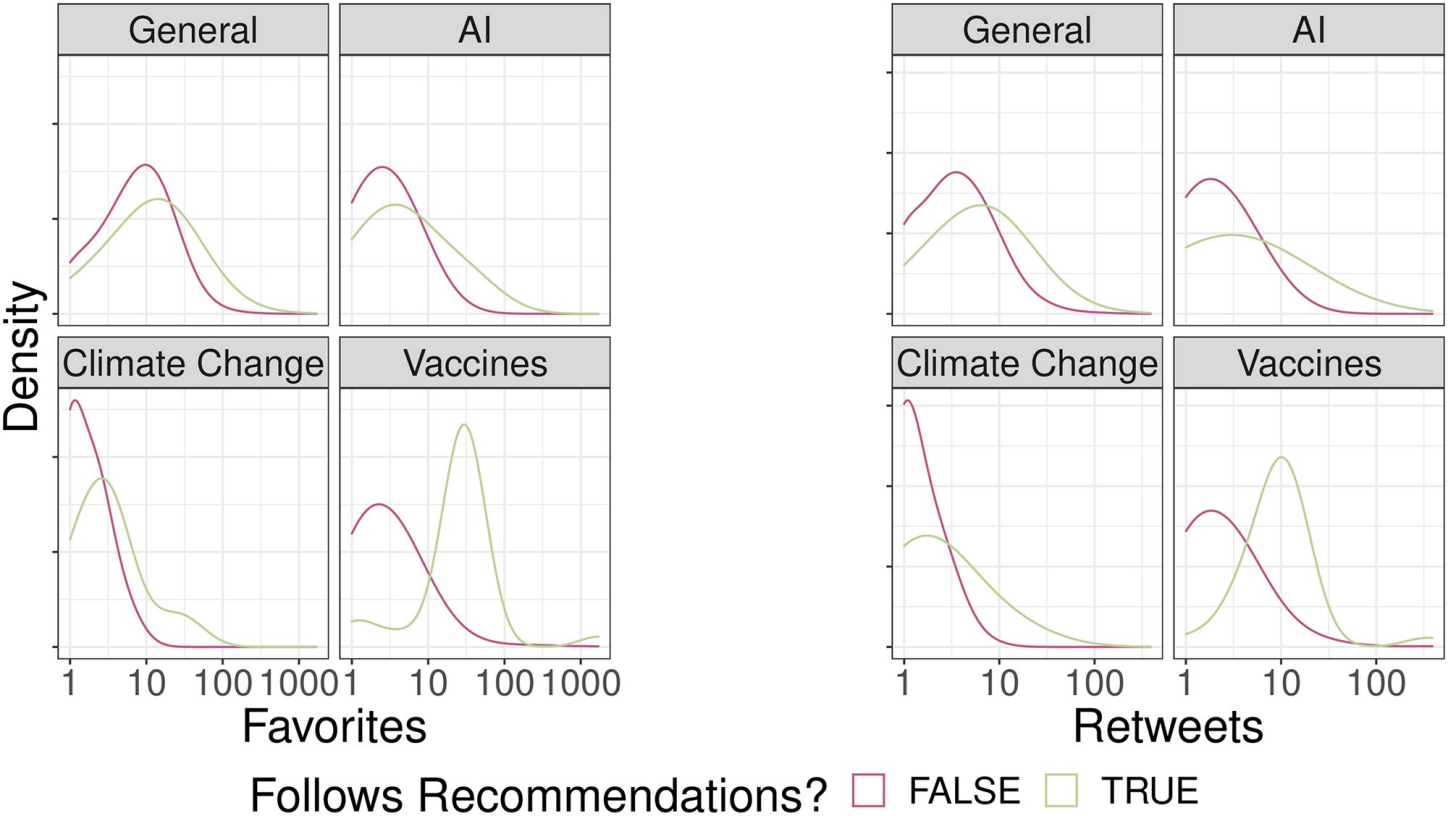
Distribution of reactions on Twitter. Kernel density estimates of Favourites (left) and Retweets (right) for each topic.

Fig 3 shows the distribution of engagement (favourites and retweets) for both groups of posts. We note that all the distributions display right-skewness (i.e., large positive deviations in terms of engagement) and that the median engagement is higher for posts following the recommendations. Such evidence is, in many instances, confirmed by the values reported in Table 4 and by the results of the non-parametric statistical tests (Mann-Whitney U test), reported in Supplementary Information. Assuming a significance level of 5%, the results of the test indicate a statistically significant difference between the favourites and retweets distributions of posts following the recommendations and posts not following the recommendations for all topics except Artificial Intelligence. Moreover, when statistically significant, the percentage change in median engagement of posts following the recommendations is +100% for Climate Change (+1 Favourite), +85.71% for General (+6 Favourites) and +2500% for Vaccines (+25 Favourites). Similarly, the percentage change in median engagement in terms of Retweets is increasing for the three aforementioned topics (see Table 5 for details).

**Table 4:** (Twitter) test statistics and p-values for the Mann-Whitney U test used for comparing the distributions of posts following and not following the recommendations.

		General	Artificial Intelligence	Climate Change	Vaccines
Test statistic	Favourites	196654	221	17924	3072
	Retweets	213286	238	15426	3147
p-value	Favourites	<0.005	0.057	<0.005	<0.005
	Retweets	<0.005	0.116	<0.005	<0.005

**Table 5:** (Twitter) percentage changes in terms of engagement with posts. The increase/decrease is reported within parentheses.

	General	Artificial Intelligence	Climate Change	Vaccines
Ratio Median Favourites	85.71% (7→13)	100% (2→4)	100% (1→2)	2500% (1→26)
Ratio Median Retweets	175% (2→5.5)	100% (1→2)	Inf (0→1)	Inf (0→9)

#### Facebook

**Fig 4:**
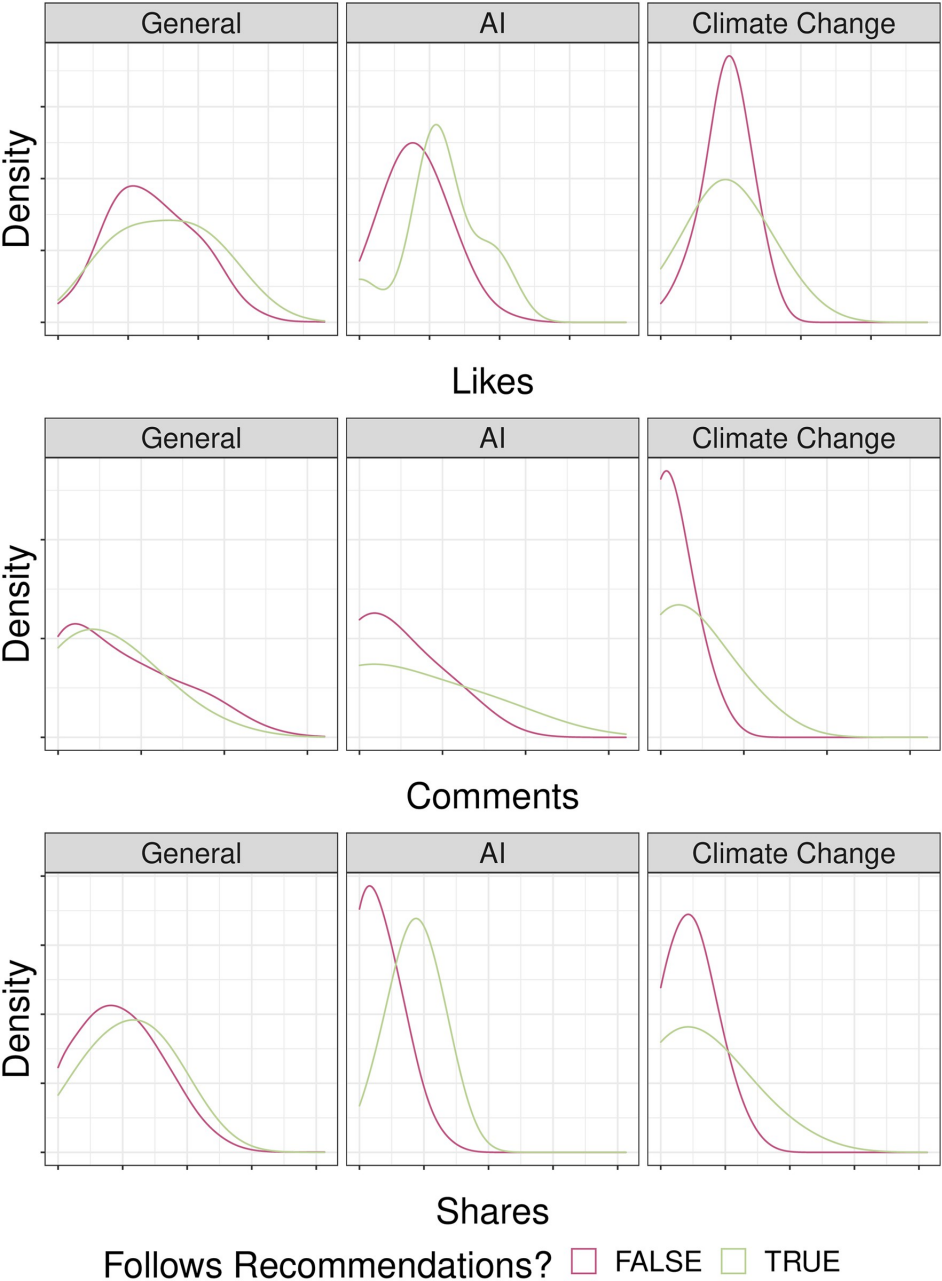
Distribution of reactions on Facebook. Kernel density estimates of Likes (top), Comments (centre) and Shares (bottom) for each of the four topics on Facebook. The topic Vaccines is missing due to the limited number of posts.

A similar analysis was performed for Facebook. Fig 4 shows the distribution of engagement (likes, comments and shares) for both groups of posts. We may notice that all the distributions display right-skewness (i.e., large deviations in terms of engagement) and that the median engagement in certain instances is higher for posts following the recommendations, as reported in Supplementary Information. Table 6 shows the results of the non-parametric statistical tests (Mann-Whitney U test). Assuming a significance level of 5%, we do not observe a statistically significant difference in the distribution of engagement for the case of Climate Change, while this difference is significant for the other two topics, except for the case of comments to Artificial Intelligence posts and for the case of shares to General posts. When statistically significant, the percentage change of median engagement of posts following the recommendations is +136.36% for Artificial Intelligence (+7.5 likes) and +100% for General (+19 Likes). Always considering only statistically significant results, the increase of the median number of comments per post changes from zero to one comment for General while the increase of the median number of shares per post changes from 0 to 5.5 shares for Artificial Intelligence (see Table 7 for details).

**Table 6:** (Facebook) test statistics and p-values for the Mann-Whitney U test used for comparing the distributions of posts following and not following the recommendations.

		General	Artificial Intelligence	Climate Change
Test statistic	Likes	131276	263.5	1758.5
	Comments	136207	397.5	1779.5
	Shares	138266	217.5	2170
p-value	Likes	0.017	0.03	0.36
	Comments	0.029	0.21	0.134
	Shares	0.0850	0.004	0.347

**Table 7:** (Facebook) percentage changes in terms of engagement with posts. The increase/decrease is reported within parentheses.

	General	Artificial Intelligence	Climate Change
Ratio Median Likes	100% (19→38)	136.36% (5.5→13)	0% (8→8)
Ratio Median Comments	Inf (0→1)	0% (0→0)	0% (0→0)
Ratio Median Shares	66.67% (6→10)	Inf (0→5.5)	-50% (2→1)

To sum up, our results show clues of a positive association between the adoption of the recommendations and engagement, particularly on Twitter. On Facebook, the topic of Vaccines was excluded from the analysis due to the small number of posts (N = 5), while for what concerns posts about Climate Change the results did not reach statistical significance. For General content, we had somewhat inconsistent results, with posts achieving more median likes, but fewer median comments when adopting the recommendations, while the difference in shares did not reach statistical significance. Despite these weaknesses, the results in terms of engagement are overall positive and encouraging. The recommendations proved to be particularly effective on Facebook for the topic of Artificial Intelligence, for which likes more than doubled, and shares increased more than five times.

#### Combined effect of recommendations

After having tested the overall usefulness of the proposed recommendations in terms of engagement, we aim at measuring their impact (either in groups or singularly) on posts. To perform this task, we rely on negative-binomial regression as this type of tool is well suited to handle count data with over dispersion [42]. Similarly to what can be found in the literature [43, 44], we regress our engagement variables—retweets and favourites for Twitter, and likes and shares for Facebook—on our set of recommendations and on a further set of control variables. Please note that Facebook comments are excluded from this analysis due to the presence of algorithmic convergence issues.

We propose two alternative specifications. In the first specification, we regress our engagement measures on four variables (Trustworthiness and Scientific Rigour (R), Presentation and Style (S), Impact on Society (SI) and 3Ts (T)) obtained by computing the number of corresponding recommendations adopted in each post:

$$y_{i}=\alpha +\beta_{1}R_{i}+\beta_{2}S_{i}+\beta_{3}SI_{i}+\beta_{4}T_{i}+Z_{i}\gamma +\epsilon_{i}\;.$$

This way, we are able to assess whether implementing recommendations from any of the conceptual areas presented in the section entitled Recommendations for Quality and Effectiveness can be associated with an improvement in engagement. The second specification takes into account the set of all recommendations in the form of dummy variables, **X**_*i*_:

$$y_{i}=\alpha +X_{i}\beta +Z_{i}\gamma +\epsilon_{i}\;.$$

To improve unconditional estimation (i.e., the risk is that of having some coexisting confounding effects) we also include a set of control variables in both of our specifications. For what concerns the set of control variables **Z**_*i*_, we consider: page fixed effects (one for each social media account in our dataset), topic fixed effects (whether the topic is General, Artificial Intelligence, Climate Change or Vaccines), day-of-the-week fixed effects and content language (whether Italian or English). As a further robustness check we also implement log-linear OLS regression and quasipoisson regression. We do not observe substantial differences.

**Fig 5:**
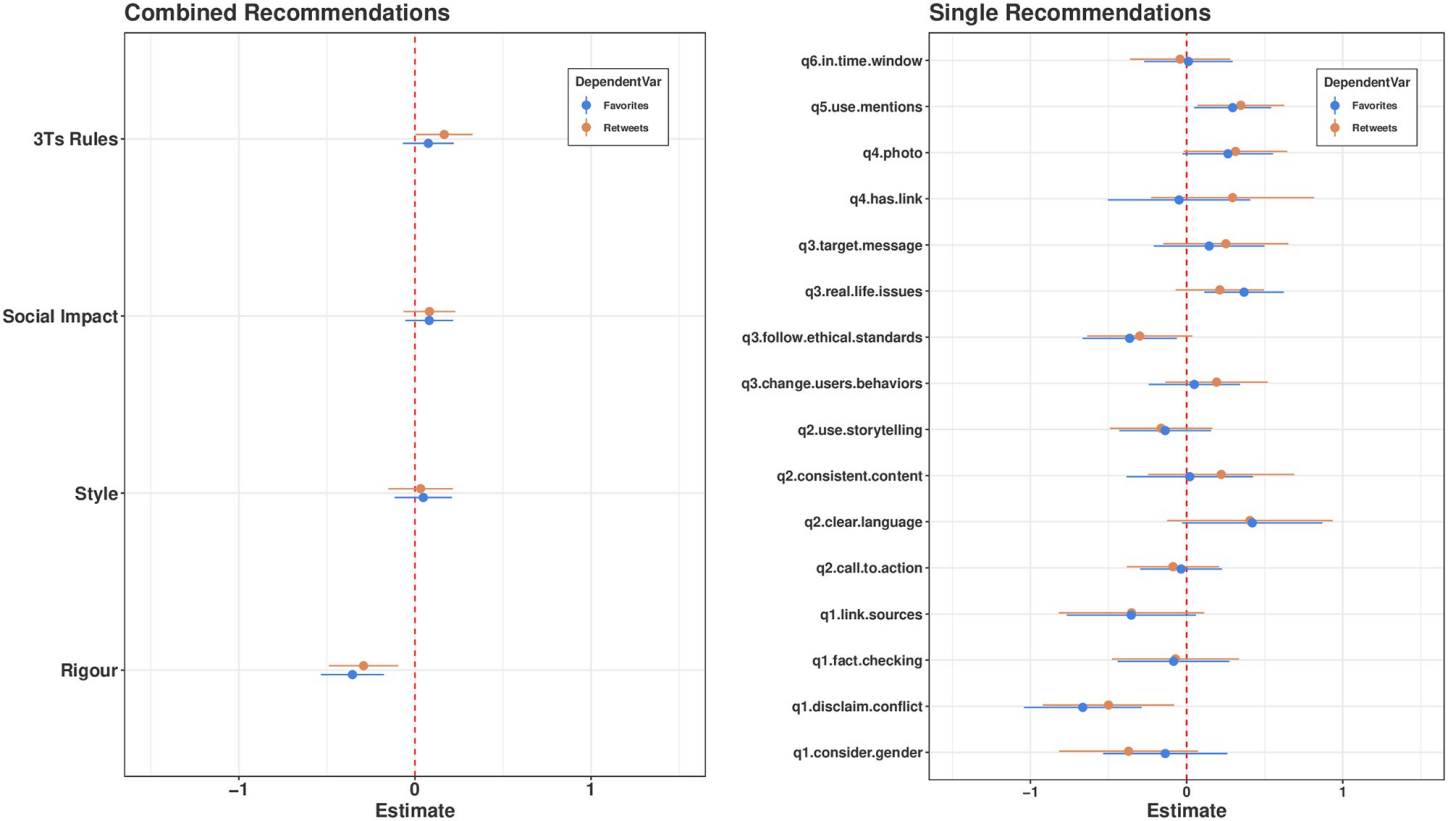
Point estimates and 95% confidence interval of negative-binomial regressions coefficients (Twitter). Left Panel: Coefficient estimates obtained by regressing favourites (in blue) and retweets (in red) on the number of recommendations adopted for each leading principle. Right panel: Coefficient estimates obtained by regressing favourites and retweets on each recommendation.

Fig 5 displays regression estimates for Twitter along with their 95% confidence intervals. By looking at the combined-recommendations results (left panel), we may notice that the sign associated to the Rigour principle is negative and significant for both favourites and retweets, while the 3Ts-Rule coefficient is mildly positive and significant for retweets. Social-Impact and Style recommendations do not seem to significantly affect engagement in aggregate terms. Focusing on single-recommendation results (right panel), we notice that the conflict-of-interest recommendation seems to be the one driving the negative impact of Rigour on both Twitter engagement measures, while the use-mentions and photo recommendations drive the positive impact of the 3Ts rules on retweets.

**Fig 6:**
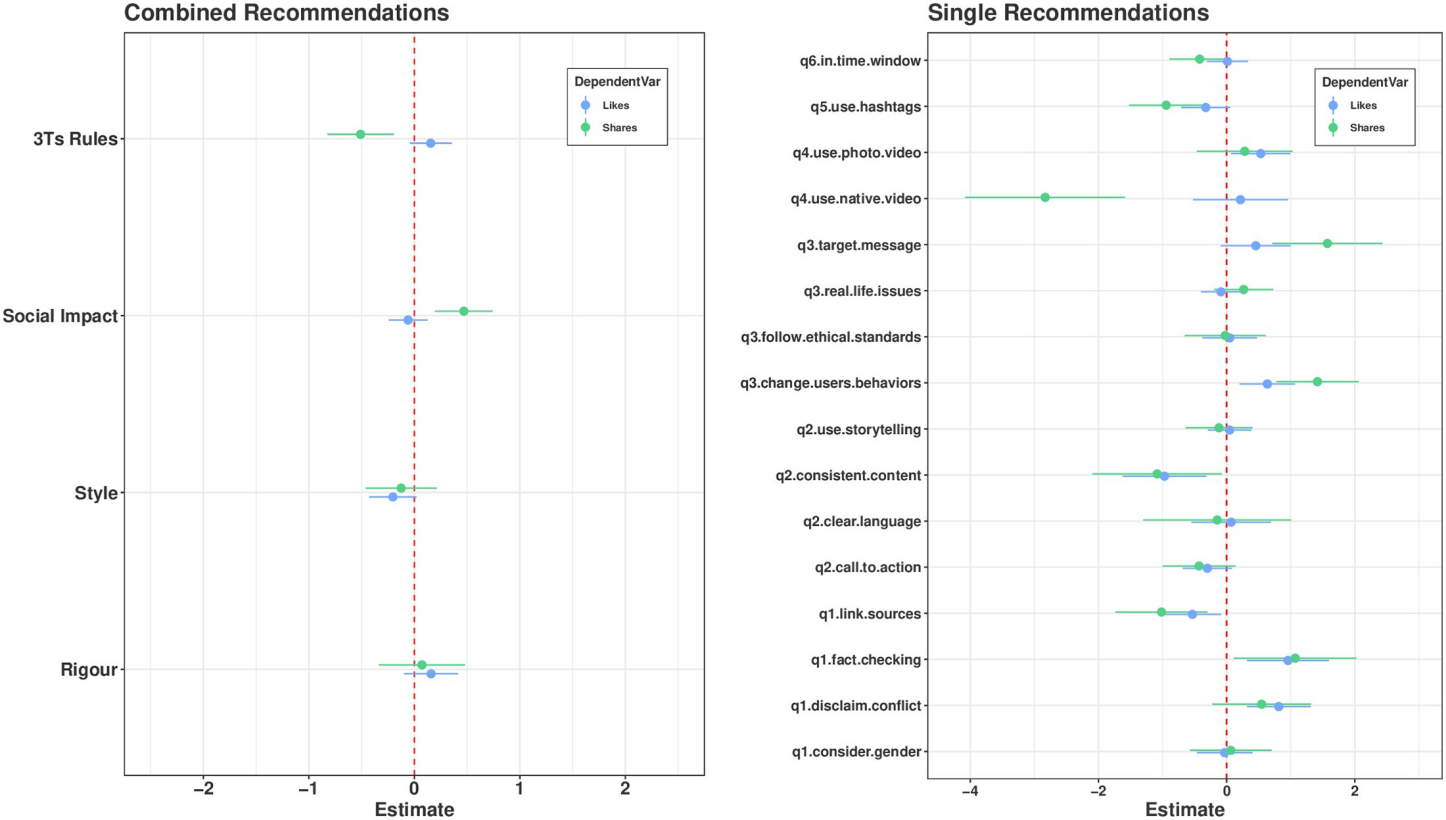
Point estimates and 95% confidence interval of negative-binomial regressions coefficients (Facebook). Left Panel: Coefficient estimates obtained by regressing likes (in blue) and shares (in green) on the number of recommendations adopted for each leading principle. Right panel: Coefficient estimates obtained by regressing likes and shares on each recommendation.

Fig 6 reports the regression estimates for Facebook. Looking at the aggregated-recommendation results (left panel), we notice that the number of shares is positively and significantly associated with the Social-Impact principle and negatively and significantly associated with the 3Ts-Rule principle. On the contrary, likes do not seem to vary according to any of the principles considered. Focusing on the single-recommendation results (right panel), we notice that the change-in-users-behaviour and target-message recommendations seem to be those driving the positive relationship between shares and social impact, while the use of native videos and hashtags seems to be the main source of the negative relationship between 3Ts and shares. Furthermore, we notice that the fact-checking recommendation is positively and significantly associated with both engagement measures, while link-sources and consistent content recommendations are negatively and significantly associated with both engagement measures.

Summing up our results about quality recommendations, we find that Trustworthiness and Scientific Rigour, especially in the form of disclaim-conflict recommendations, does not seem to be repaid by users in terms of Twitter engagement. On Facebook, we find that Impact on Society recommendations pay back in terms of shares in aggregate terms. Also fact checking seems to pay back in terms of engagement, while the opposite is true for the link-sources recommendation.

Coming to the purpose of testing the effectiveness of 3Ts rules, our results are mixed. On the one hand, the case of retweets corroborates our expectations, especially if we consider the coefficients associated with use-mentions and photos. On the other hand, we find that 3Ts rules seem to be counteractive for what concerns Facebook shares. In particular, the use of native videos and hashtags seem to be negatively associated with the number of shares.

Although the recommendations are crafted taking into account the features of the social media platform in which they will be implemented, it is likely that something generating more engagement on one platform may fail on another due to the different segments of users involved and to the discrepancies in the design and recommendation algorithms implemented.

## Discussion

Our results show an overall positive increase in engagement, which is statistically significant when compared to social media content for which the recommendations were not considered. The percentage change in the median engagement was particularly pronounced on Twitter, with an approximate improvement of at least two times in favourites and retweets. Moreover, we evaluated the effect of single recommendations in terms of engagement using a negative-binomial regression model. The results show that the recommendations have positive effects on Twitter, which mainly leverage on platform-specific aspects (e.g., involving other users in the discussion, or including photos). On Facebook, this may be observed for recommendations that focus on societal issues and trustworthiness (e.g., promoting virtuous behaviours, or presenting reliable content). To maximise the chances of adoption of the recommendations in the relevant communities, we applied a co-design approach and avoided any top-down solution. Here we discuss the results, contributions, and limitations of this study.

### Quality and effectiveness

Evaluating the helpfulness of the recommendations was limited by the nontrivial challenge of first defining and then measuring quality in quantitative terms. The recommendations proposed in this paper integrate 12 qualitative indicators that can be grouped into three main conceptual areas: trustworthiness and scientific rigour, presentation and style, and the societal impact of the communication [30]. These indicators reflect the perspectives of different stakeholders (e.g., journalists, scientists, general public) on what constitutes “quality” in science communication. Clearly, these definitions do not come without drawbacks. Trustworthiness and scientific rigour, for example, cannot be guaranteed by simply including a link to a reliable source; clarity is hard to define; the use of storytelling, while making science communication more appealing, often limits the capacity of conveying the complexity of science, with the risk of misleading the public; impact on society is hard to assess. Nonetheless, the main aim of these recommendations is to raise awareness and understanding among communication practitioners and experts, and engage them in a critical reflection on these aspects. Based on the feedback received from the participants to our experiment, the recommendations fulfil this function successfully.

Similarly, measuring effectiveness is not straightforward, since being *effective* usually depends on the goal that has been set for the communication. Effectiveness may be determined on different levels, from the ability to educate and raise awareness, to make the public interested in and supportive towards science, to even change individuals’ behaviour (e.g., quit smoking, reduce alcohol drinking, have a healthy lifestyle). In all these cases, science communication would have valuable effects. However, this is hardly measurable quantitatively, especially when analysing social media data, since one should trace a causality link between users’ exposure to online information and their offline behaviour. For these reasons, in this paper we focused on indicators of engagement that can be easily derived from the available data, such as the number of retweets, likes, comments, and shares. However, engagement can be considered as a good proxy of outreach and appreciation and, thus, of effectiveness.

### Results

The analysis on Twitter shows that the posts following our recommendations achieved a significant higher median engagement than their counterpart produced in the same period. Only for tweets from accounts specialised in Artificial Intelligence this advantage did not reach a statistically significant level. This can be due to the limited number of posts created following the recommendations (N = 14). On the other hand, Artificial Intelligence was also the topic that achieved the best results on Facebook, a platform where the topic of Vaccines was scarcely represented in our sample, and the topic Climate Change did not reach statistical significance. Results for Facebook are similar and confirm a statistically significant advantage in terms of engagement derived by the adoption of the recommendations, except for climate change accounts.

To assess the relationship between recommendations adoption and engagement, we also considered the fact that not all recommendations were necessarily applied in each post. Therefore, we ran regressions to test the impact of recommendations while controlling for possible confounding effects. We noticed that *Trustworthiness and Scientific Rigour* recommendations do not pay back in terms of engagement on Twitter, while *Impact on Society* recommendations seem to be effective to promote shares. Moreover, we found mixed evidence about the effectiveness of the 3Ts’ rule. In particular, we observed that the adoption of some recommendations (e.g., use-mentions and photos) are positively associated with retweets and favourites on Twitter, while others such as the use of hashtags are negatively associated with shares on Facebook.

Throughout the whole experiment, the role of the participants was key, not only in following our recommendations, but also in providing comments and suggestions for their further refinement and improvement. For most respondents the recommendations were easy-to-adopt and proved useful for communicating science in social media. Some recommendations were rarely applied (e.g., posting original videos) because of their requirement in terms of resources (e.g., time, skills). In this regard, the pursuit of quality and effectiveness inevitably collide with the business model and affordability of science communication in social media, that is too often considered secondary to more traditional channels in terms of investment.

### Limitations

The experiment may have been affected by the unfortunate outbreak of COVID-19. While the pandemic certainly contributed to highlight the key role of science communication, it has also called the public attention upon the new coronavirus, making the topics selected in this paper (especially climate change and artificial intelligence) less important. In addition, the dominance of the COVID-19 topic and the exceptional engagement that this generated online prevented us to compare posts adopting the recommendations with posts of the same accounts in the period before the experiment (and thus before the pandemic), as in our original plans. Comparing the two groups of posts (following/not following recommendations) in the same time window may be subject to biases that may hinder the effectiveness of such measurement. Unfortunately, we cannot claim that the sample of participants joining the experiment is representative of the entire Europe. Due to the high fragmentation of the science communication field, it is hard to obtain a representative sample of the entire community. The field is evolving very rapidly, and is constituted by a heterogeneous set of actors [27]. However, the final set of participants include a wide variety of communication practitioners with different perspectives and backgrounds, such as industries, organisations, magazines/publications, science journalists, experts, and universities. Furthermore, the 3Ts recommendations were developed on the basis of a novel dataset of 498 Facebook pages and 661 Twitter accounts across seven European countries, for a total of 2M posts [27], offering for the first time large-scale data-driven insights about science communication on social media platforms.

## Conclusions

In conclusion, our work shows the goodness and potential of a data-driven approach in fostering quality and effective science communication on social media. Overall, the adoption of our recommendations results in positive outcomes in terms of engagement, showing that social media can be an effective channel to promote engagement with science (RQ1) and that quality content is able to involve users on social media (RQ2). Moreover, the experiment presented in this paper highlights the many benefits offered by the reciprocal collaboration between scholars and communication practitioners. These results may pave the way for further research developments. Future research will be devoted to replicate this study with a larger sample of participants in a more regular setting, and to investigate possible drivers of users’ response to quality content. In the context of the ever-evolving online environment, such work is not only desirable, but necessary to provide empirical evidence to tailoring effective science communication strategies.

## Supporting information

S1 Table Breakdown of recommendations. Recommendations by thematic area.
(PDF)

S2 Table Summary statistics (Twitter). Summary statistics for the distribution of Favourites and Retweets.
(PDF)

S3 Table Summary statistics (Facebook). Summary statistics for the distribution of likes, comments and shares for General (G), Artificial Intelligence (AI), and Climate Change (CC) posts.
(PDF)

S4 Table Regression table (Twitter)—Aggregated recommendations. (PDF)

S5 Table Regression table (Twitter)—Single recommendations. (PDF)

S6 Table Regression table (Facebook)—Aggregated recommendations. (PDF)

S7 Table Regression table (Facebook)—Single recommendations. (PDF)
