# Supplementary material for: Promoting engagement with quality communication in social media
Source: PLoS One. 2022 Oct 13;17(10):e0275534. doi: 10.1371/journal.pone.0275534 (PMC9560150; doi:10.1371/journal.pone.0275534)
Supplement: S1 Table — Recommendations by thematic area. (PDF) [file pone.0275534.s001.pdf]

	Twitter	Facebook
Trustworthiness and scientific rigour	fact_checking<br>link_sources<br>disclaim_conflict<br>consider_gender	fact_checking<br>link_sources<br>disclaim_conflict<br>consider_gender
Presentation and style	clear_language<br>consistent_content<br>use_storytelling<br>call_to_action	clear_language<br>consistent_content<br>use_storytelling<br>call_to_action
Impact on society	real_life_issues<br>change_users_behaviours<br>target_message<br>follow_ethical_standards	real_life_issues<br>change_users_behaviours<br>target_message<br>follow_ethical_standards
3Ts	photo<br>has_link<br>use_mentions<br>in_time_window	use_photo_video<br>use_native_video<br>use_hashtags<br>in_time_window

Table S1. Recommendations by thematic area.
