# Supplementary material for: Promoting engagement with quality communication in social media
Source: PLoS One. 2022 Oct 13;17(10):e0275534. doi: 10.1371/journal.pone.0275534 (PMC9560150; doi:10.1371/journal.pone.0275534)
Supplement: S2 Table — Summary statistics for the distribution of Favourites and Retweets. (PDF) [file pone.0275534.s002.pdf]

	Follows Recommendations?	Mean Favourites	Mean Retweets	Median Favourites	Median Retweets	Skewness Favourites	Skewness Retweets
G	No	10.3	4.15	7	2	7.01	9.14
	Yes	19.1	8.25	13	5.5	6.56	4.80
AI	No	2.94	1.40	2	1	1.01	1.3
	Yes	9	3.71	4	2	1.36	1.25
CC	No	1.15	0.615	1	0	2.31	2.89
	Yes	4.89	2.24	2	1	2.79	2.88
V	No	6.04	2.34	1	0	21.0	22.1
	Yes	72.8	18.2	26	9	5.30	5.26

Table S2. (Twitter) Summary statistics for the distribution of Favourites and Retweets.
