# Supplementary material for: Promoting engagement with quality communication in social media
Source: PLoS One. 2022 Oct 13;17(10):e0275534. doi: 10.1371/journal.pone.0275534 (PMC9560150; doi:10.1371/journal.pone.0275534)
Supplement: S3 Table — Summary statistics for the distribution of likes, comments and shares for General (G), Artificial Intelligence (AI), and Climate Change (CC) posts. (PDF) [file pone.0275534.s003.pdf]

	Follows Recomm.?	Mean Likes	Mean Comments	Mean Shares	Median Likes	Median Comments	Median Shares	Skewn. Likes	Skewn. Comments	Skewn. Shares
G	No	68.2	9.80	33.9	19	0	6	17.6	15.4	38.5
	Yes	88.2	5.45	27.4	38	1	10	2.31	5.23	2.63
AI	No	10.4	0.85	1.25	5.5	0	0	4.87	4.31	3.88
	Yes	28.4	4	7.12	13	0	5.5	1.30	1.85	0.89
CC	No	9.63	0.143	2.97	8	0	2	1.34	4.68	2.84
	Yes	17.4	0.636	7.33	8	0	1	2.88	4.37	3.82

Table S3. (Facebook) Summary statistics for the distribution of likes, comments and shares for General (G), Artificial Intelligence (AI), and Climate Change (CC) posts.
