# Supplementary material for: Promoting engagement with quality communication in social media
Source: PLoS One. 2022 Oct 13;17(10):e0275534. doi: 10.1371/journal.pone.0275534 (PMC9560150; doi:10.1371/journal.pone.0275534)
Supplement: S4 Table — (PDF) [file pone.0275534.s004.pdf]

	Dependent variable:					
	log(retweet.count + 1)	retweet.count		log(favourite.count + 1)	favourite.count	
	OLS	*quasipoisson*	*negative binomial*	*OLS*	*quasipoisson*	*negative binomial*
	(1)	(2)	(3)	(4)	(5)	(6)
Rigour	−0.268***	−0.733***	−0.292***	−0.333***	−1.010***	−0.355***
	(0.084)	(0.092)	(0.100)	(0.088)	(0.085)	(0.091)
Style	−0.017	0.094	0.032	−0.037	0.165	0.048
	(0.074)	(0.104)	(0.093)	(0.078)	(0.102)	(0.083)
Social.Impact	0.113*	0.088	0.083	0.084	0.093	0.081
	(0.063)	(0.082)	(0.075)	(0.067)	(0.083)	(0.069)
3Ts	0.141**	0.233**	0.166**	0.105	0.173**	0.076
	(0.067)	(0.092)	(0.082)	(0.071)	(0.087)	(0.073)
Lang_it	0.330	0.322	0.449	0.502**	0.224	0.529**
	(0.238)	(0.325)	(0.287)	(0.251)	(0.276)	(0.250)
Constant	0.836	1.505	0.152	1.296	2.907**	0.764
	(0.770)	(1.155)	(0.929)	(0.810)	(1.241)	(0.837)
θ			2.076***			2.159***
			(0.281)			(0.243)
Page F.E.	YES	YES	YES	YES	YES	YES
Topic F.E.	YES	YES	YES	YES	YES	YES
Day of the Week F.E.	YES	YES	YES	YES	YES	YES
Observations	240	240	240	240	240	240
R^2	0.584			0.678		
Adjusted R^2	0.520			0.629		
Log Likelihood			−609.538			−769.446
Akaike Inf. Crit.			1,285.076			1,604.893
Residual Std. Error (df = 207)	0.726			0.763		
F Statistic (df = 32; 207)	9.086***			13.646***		
Note:						*p<0.1; **p<0.05; ***p<0.01

Table S4. (Twitter) Regression Table - aggregated recommendations.
