# Supplementary material for: Promoting engagement with quality communication in social media
Source: PLoS One. 2022 Oct 13;17(10):e0275534. doi: 10.1371/journal.pone.0275534 (PMC9560150; doi:10.1371/journal.pone.0275534)
Supplement: S6 Table — (PDF) [file pone.0275534.s006.pdf]

	Dependent variable:					
	log(likes + 1)	likes		log(shares + 1)	shares	
	OLS	*quasipoisson*	*negative binomial*	*OLS*	*quasipoisson*	*negative binomial*
	(1)	(2)	(3)	(4)	(5)	(6)
Rigour	0.072	−0.230	0.159	−0.067	−0.383	0.072
	(0.136)	(0.177)	(0.130)	(0.176)	(0.240)	(0.208)
Style	−0.148	−0.022	−0.203*	−0.086	0.011	−0.125
	(0.122)	(0.152)	(0.114)	(0.158)	(0.181)	(0.171)
Social.Impact	−0.002	−0.101	−0.059	0.273**	0.289**	0.469***
	(0.101)	(0.112)	(0.093)	(0.130)	(0.134)	(0.139)
3Ts	0.094	−0.034	0.155	−0.464***	−0.543***	−0.510***
	(0.109)	(0.116)	(0.101)	(0.141)	(0.164)	(0.160)
Lang_it	0.648	0.471	0.768	−0.679	−0.824	−1.205
	(0.986)	(2.867)	(0.964)	(1.275)	(4,809.290)	(48,595,464.000)
Constant	1.377	2.021	1.239	2.123	2.669	2.173
	(1.159)	(3.058)	(1.125)	(1.499)	(4,809.290)	(48,595,464.000)
θ			2.069***			0.954***
			(0.284)			(0.148)
Page F.E.	YES	YES	YES	YES	YES	YES
Topic F.E.	YES	YES	YES	YES	YES	YES
Day of the Week F.E.	NO	NO	NO	NO	NO	NO
Observations	116	116	116	116	116	116
R^2	0.755			0.626		
Adjusted R^2	0.706			0.552		
Log Likelihood			−495.212			−373.065
Akaike Inf. Crit.			1,030.425			786.129
Residual Std. Error (df = 96)	0.787			1.018		
F Statistic (df = 19; 96)	15.552***			8.473***		
Note:						*p<0.1; **p<0.05; ***p<0.01

Table S6. (Facebook) Regression Table - aggregated recommendations.
