# Supplementary material for: Promoting engagement with quality communication in social media
Source: PLoS One. 2022 Oct 13;17(10):e0275534. doi: 10.1371/journal.pone.0275534 (PMC9560150; doi:10.1371/journal.pone.0275534)
Supplement: S7 Table — (PDF) [file pone.0275534.s007.pdf]

	Dependent variable:					
	log(likes + 1)	likes		log(shares + 1)	shares	
	OLS	*quasipoisson*	*negative binomial*	*OLS*	*quasipoisson*	*negative binomial*
	(1)	(2)	(3)	(4)	(5)	(6)
q1.consider.gender	−0.153	−0.680**	−0.030	0.119	−0.346	0.065
	(0.250)	(0.336)	(0.221)	(0.314)	(0.353)	(0.325)
q1.disclaim.conflict	0.588**	0.994**	0.813***	0.289	0.744	0.547
	(0.284)	(0.391)	(0.253)	(0.357)	(0.612)	(0.392)
q1.fact.checking	0.980***	1.758***	0.957***	0.664	1.542**	1.070**
	(0.372)	(0.644)	(0.326)	(0.467)	(0.751)	(0.488)
q1.link.sources	−0.446*	−0.984***	−0.534**	−0.781**	−1.243***	−1.016***
	(0.259)	(0.278)	(0.227)	(0.325)	(0.338)	(0.367)
q2.call.to.action	−0.291	−0.143	−0.300	−0.436	−0.488	−0.431
	(0.226)	(0.229)	(0.196)	(0.284)	(0.299)	(0.290)
q2.clear.language	0.072	0.280	0.071	−0.006	0.384	−0.147
	(0.354)	(0.754)	(0.317)	(0.445)	(1.207)	(0.588)
q2.consistent.content	−0.799**	−1.084*	−0.970***	−0.698	−1.223	−1.084**
	(0.385)	(0.627)	(0.333)	(0.484)	(0.742)	(0.515)
q2.use.storytelling	−0.011	0.081	0.047	−0.210	−0.077	−0.118
	(0.194)	(0.276)	(0.174)	(0.244)	(0.297)	(0.265)
q3.change.users.behaviours	0.549**	−0.200	0.636***	0.879***	0.751*	1.417***
	(0.250)	(0.368)	(0.220)	(0.314)	(0.404)	(0.327)
q3.follow.ethical.standards	0.009	−0.206	0.049	−0.074	−0.003	−0.022
	(0.248)	(0.257)	(0.217)	(0.311)	(0.349)	(0.322)
q3.real.life.issues	−0.122	0.217	−0.088	−0.018	0.378	0.267
	(0.183)	(0.202)	(0.160)	(0.230)	(0.273)	(0.235)
q3.target.message	0.235	0.123	0.455	0.861**	1.559*	1.573***
	(0.302)	(0.739)	(0.278)	(0.380)	(0.897)	(0.439)
q4.use.native.video	0.092	−0.911**	0.216	−2.482***	−2.455***	−2.832***
	(0.452)	(0.425)	(0.379)	(0.567)	(0.857)	(0.637)
q4.use.photo.video	0.334	0.536	0.533**	0.146	0.267	0.283
	(0.265)	(0.359)	(0.237)	(0.333)	(0.568)	(0.381)
q5.use.hashtags	−0.148	−0.522*	−0.325*	−0.647**	−1.015***	−0.943***
	(0.222)	(0.305)	(0.197)	(0.279)	(0.371)	(0.296)
q6.in.time.window	−0.058	−0.123	0.013	−0.184	−0.391	−0.420*
	(0.187)	(0.202)	(0.164)	(0.235)	(0.237)	(0.242)
Lang_it	1.838*	3.086	2.046**	0.869	2.255	1.736
	(1.065)	(2.769)	(0.973)	(1.337)	(4,216.490)	(2,096,497.000)
Constant	1.004	0.517	0.769	1.563	0.231	0.893
	(1.231)	(3.166)	(1.121)	(1.546)	(4,216.491)	(2,096,497.000)
θ			2.666***			1.332***
			(0.384)			(0.220)
Page F.E.	YES	YES	YES	YES	YES	YES
Topic F.E.	YES	YES	YES	YES	YES	YES
Day of the Week F.E.	NO	NO	NO	NO	NO	NO
Observations	116	116	116	116	116	116
R^2	0.801			0.714		
Adjusted R^2	0.728			0.609		
Log Likelihood			−481.522			−357.189
Akaike Inf. Crit.			1,027.045			778.377
Residual Std. Error (df = 84)	0.758			0.951		
F Statistic (df = 31; 84)	10.924***			6.780***		
Note:						*p<0.1; **p<0.05; ***p<0.01

Table S7. (Facebook) Regression Table - single recommendations.
